# Residential Transitions in Supported Living: Experiences of Adults With Intellectual Disabilities and Parents

**DOI:** 10.1111/jar.70225

**Published:** 2026-04-07

**Authors:** Kim Ulvin, Laila Tingvold, Karina Aase, Siv Fladsrud Magnussen

**Affiliations:** ^1^ Centre for Care Research East Norwegian University of Science and Technology (NTNU) Gjøvik Norway; ^2^ Centre for Resilience in Healthcare SHARE, Faculty of Health Sciences University of Stavanger Stavanger Norway

**Keywords:** housing, parents, residential transitions, supported living

## Abstract

**Background:**

This article explores how adults with intellectual disabilities and a corresponding parent group experience residential transitions to and within co‐located supported housing in Norway.

**Method:**

We conducted 12 semi‐structured interviews and a supplementary focus group interview and analysed the data using reflexive thematic analysis.

**Results:**

Two overarching themes were developed, (1) First, find a place: a long and uncertain search marked by scarce options, opaque entitlements, and unclear responsibilities. (2) Settling in(to) a constructed home: evolving negotiation around role and agency, negotiations over who has to adjust to whom, and coping with aspirations and limitations related to future mobility amid the immersive potential of co‐located supported housing.

**Conclusion:**

Residential transitions shape more than a single move. They influence perspectives on future residential mobility and aspirations for living arrangements. Improved communication about rights, eligibility thresholds, and realistic timelines may reduce disillusionment and improve experiences with transitions.

## Lay Summary

- Finding a place to live often takes years. Once a place is found, the move happens quickly, but settling in takes a long time.
- To improve the experiences of persons with intellectual disabilities and parents during the housing search and move, the municipality should clearly communicate the rights to housing and services and explain when these two are linked.
- The municipality should work to communicate more clearly its role and responsibility, highlight the different types of municipal housing, and be more attentive to residential mobility for persons who already live in co‐located supported housing.
- Negative experiences searching for and settling into co‐located supported housing can limit persons with intellectual disabilities' aspirations and make parents less likely to see the upside of moving in the future.

## 1 Introduction

Research on the experiences related to residential transitions to and within independent community living arrangements for adults with intellectual disabilities is limited, and likewise are studies exploring the experiences of their parents (Grey et al. 2015; Walker et al. 2025). Parents of adults with intellectual disabilities often remain actively involved, assuming both formal and informal roles, and provide vital support resources far beyond the first move out of the family home, frequently remaining engaged as long as they are capable (Codd and Hewitt 2021; Vereijken et al. 2024).

The social, cultural, and broader national institutional contexts, including the welfare system and entitlement schemes, influence and shape care approaches, living arrangements, and opportunities for residential mobility for individuals with intellectual disabilities and shape their residential transition experience (Bigby et al. 2017; Skagestad et al. 2023; Zaviršek and Fischbach 2023). According to Šiška and Beadle‐Brown (2023), the common living arrangements for persons with intellectual disabilities in the European Union are residing with family or living independently in larger co‐located or clustered arrangements with six or more persons, with Sweden as the exception. Although Scandinavian countries share a similar welfare‐state model and a commitment to the UN Convention on the Rights of Persons with Disabilities (UN 2006) as an aspirational policy framework, there are differences in their policy and regulatory approaches to housing and support for persons with intellectual disabilities (Tøssebro 2016; Tøssebro et al. 2012). Sweden has restrictions that ensure small residential settings and was earlier in moving in a rights‐based direction, specifically for personalised care services, but also for housing (Berlin Hallrup et al. 2019; The National Board of Health and Welfare 2021; Tøssebro 2016).

In Norway, most young adults, including those with intellectual disabilities, move to independent living arrangements at a reasonably young age, comparable to the European Union (Dommermuth 2009; Ferraretto and Vitali 2023; Tøssebro and Wendelborg 2021). The most common housing option in Norway for adults with intellectual disabilities is municipally operated co‐located supported housing (Tøssebro and Wendelborg 2021). However, for over a decade the prevailing reality in terms of option and choice, as it can be related to Article 19 of the CRPD (UN 2006), regarding living arrangements for persons with intellectual disabilities in Norway, has in the research been framed as a matter of ‘take it or leave it’ as people are in practice left with only one option (Tøssebro 2016). Norwegian research has further shown that the initial transition out of the family home is often delayed (Roos and Søndenaa 2020), and there is an increased prevalence of urgent residential transitions (Astrup et al. 2019; Sandvin and Anvik 2020).

Few studies have explored the broader experiences of adults with intellectual disabilities and their families regarding residential transitions to and within supported living arrangements (Walker et al. 2025). Limited research exists on the broader experience of residential transitions, pertaining to residential mobility, the service development process encapsulating it, and its impact on the lives and outlooks of adults with intellectual disabilities and their parents. Likewise, there is scarce Norwegian research on the effects of the practical realities of the regulatory framework and disability social regulation on residential mobility and transition processes to and within independent community living arrangements, from the perspectives of persons with intellectual disabilities or their parents (Sandvin and Anvik 2020; Tøssebro 2016, 2019).

To address this gap, we conducted a qualitative study to explore the lived experiences of adults with intellectual disabilities and parents. This article examines their narrative accounts and experiences with residential transitions to and within co‐located supported housing. Our study is informed by Public Service Logic (PSL) (Osborne 2020), employs a value co‐creation lens and has an analytical focus on the user‐provider relations and the public service delivery process and outcomes (Osborne 2018, 2020). PSL posits user involvement as an intrinsic element of value creation in public services (Cui and Osborne 2022; Osborne 2018). Value co‐creation is distinguished from and extends beyond co‐production, viewing it as a dynamic, interactive relationship in which value emerges through resource integration and interaction, and value can be created or destroyed within the context of the service user's wider life experience (Grönroos 2019; Grönroos and Voima 2013; Osborne et al. 2022). To this end, our research question is:

- How do adult individuals with intellectual disabilities and a corresponding parent group experience residential transitions to and within co‐located supported housing?

### 1.1 The Norwegian Setting

A community‐based approach to accommodation and support for people with intellectual disabilities was implemented in Norway in 1991 (Tøssebro 2016). In Norway, the municipalities are legally and fiscally responsible for ensuring adequate and coordinated care services (Health and Care Services Act 2011, p. §§ 1–1, 4–1). When the extent of an individual's needs can only be met adequately through an around‐the‐clock service arrangement, it is the extent of the need for municipal care and health services that constitutes the basis for the legal right to be offered a suitable housing arrangement (Health and Care Services Act 2011, § 3‐2 a; Patients and User Rights Act 1999, § 2‐1 e; Prop. 99 L (2015–2016) 2016).

Despite the absence of a clear legal definition, the threshold for around‐the‐clock service needs effectively establishes a two‐tiered system regarding housing rights for persons with intellectual disabilities. In the case of an individual's extent of service needs not reaching the around‐the‐clock threshold, the municipalities are only obligated to guide and assist those who cannot themselves meet their needs in the housing market (The Social Housing Act 2022, §§ 1, 4, 5). This distinction is often framed in legal terms as *the right to services* and *the right to compete for scarce resources* (Health and Care Services Act 2011; Østenstad 2011; The Social Housing Act 2022). Any offer of an independent living arrangement made under the latter category is derived from the municipality's private autonomy and professional discretion (Bendixen 2024). Such offers can be made with conditions, and it is common for municipalities to link an offer of supported housing with a package of statutory services (Bendixen 2024).

Based on the regulatory framework, three distinct relationships between housing and services exist, which can be summarised as service‐derived, packaged, or decoupled (Bendixen 2024). In practice, the professional assessment of the level of services needed is multifactorial and temporally contingent, dependent on the individual's care, support, and supervision needs, as well as the functionality of the current living arrangement and the ability to acquire alternatives. Lacking any other options, the municipality has a preceptory responsibility to provide shelter (Act relating to social services 2009, § 27).

Extending the responsibility of the municipality also includes the service users' rights to self‐determination, be heard, collaborate, and individual case treatment and delivery facilitation (Health and Care Services Act 2011; Patients and User Rights Act 1999). This is further established in the national guidelines, which elaborate on the obligations to facilitate co‐creation of services and aid choice, layout, and ergonomic facilitation of the home for persons with intellectual disabilities (The Norwegian Directorate of Health 2021).

## 2 Method

### 2.1 Study Design

A qualitative research design was applied using semi‐structured interviews and complemented by a focus group interview. The study employed a collective instrumental case study strategy (Stake 1995), and the analysis was inspired by Braun and Clarke's (2022) reflexive thematic analysis. A restricted narrative strategy was adopted, primarily to organise the data chronologically and to group events and experiences into process stages (Bennett and Checkel 2015; Brinkmann and Kvale 2015, 178–181; Clark et al. 2021, 445–446; Langley 1999). This study is part of a university–municipality collaboration and was conducted within a single municipality.

### 2.2 Recruitment

Initially, information about the study was shared via the municipal service network and sent to a local third‐sector interest organisation for persons with intellectual disabilities and their families. The municipal service provider distributed an information letter about the study to adults with intellectual disabilities living in co‐located supported housing and their families. Participants were initially recruited through purposive sampling, with a snowball element, whereby parents referred other parents to participate. Adults (aged 18+) with intellectual disabilities who had experience with moving to and within co‐located supported housing, as well as parents or other family members, were eligible to participate.

There was only one parent–child dyad among the participants. Based on the parents' recommendation, no further efforts were made to increase the number of parent–child dyads.

The decision to include a focus group interview was made subsequent to a request from the secretary of a designated municipal user group council, whose members were adults with intellectual disability. The council secretary contacted the first author to express the council members' interest in sharing their experiences, perspectives, and views on home shift processes to and within co‐located supported housing, as well as on residential mobility more generally. Efforts to recruit parents to participate in separate focus group interviews were unsuccessful.

### 2.3 Participants

A total of five persons with intellectual disabilities and eight parents participated in 12 face‐to‐face, in‐depth interviews for the study. The first author conducted these interviews from November 2022 to March 2023. The interviews lasted between 42 and 100 min. In one interview, both parents took part. Four adults with intellectual disabilities participated in the focus group interview, and two of the four also participated in individual interviews.

All participants identified themselves as being of Scandinavian descent. According to their reports, the parent group had a wide variety of professional backgrounds, socioeconomic statuses, and nuclear family structures and sizes. Five parents described their adult children as falling within the mild‐to‐moderate range, whereas the remaining parents reported a higher classification in the severe range. Among the adult persons with intellectual disabilities, three reported having physical mobility limitations or sensory comorbidities. All persons had personal competence. Based on the first author's objectively limited observation and grounds for assessment, all persons with intellectual disabilities could be classified as falling in the mild to moderate range in terms of severity. Table 1 presents an overview of the participants.

**TABLE 1:** Characteristics of the participants.

Participants, in‐depth interviews, *n* = 13	
Participants, focus group interviews, *n* = 4	
Persons with intellectual disabilities and residential transition experience, *n*	7
Female	6
Male	1
Moved more than once	4
Age range (individual interviews)	(26–58, mean 31)
Age range (focus group interview)	(34–58, mean 46)
Parents of persons with intellectual disabilities with residential transition experience, *n*	8
Female	6
Male	2
Age range	(57–84, mean 71)
Married/cohabiting	5
Single	3

### 2.4 Data Collection

The interviews were semi‐structured. All interviews began with an open‐ended invitation for participants to share their stories of home shifting, encouraging them to speak freely about their experiences. The questions were framed in plain language, and the interview approach was designed to build on participants' stories. Participants were encouraged to discuss issues important to them (Brinkmann and Kvale 2015; Clark et al. 2021). The interview guide ensured that all participants were asked the same set of broad questions and aided the later stages of the interviews with the parent group. The format allowed flexibility: initially, the questions were narratively exploratory, enabling discussion of issues not predetermined, and participants were encouraged to begin and develop their stories in ways that made sense to them. When they paused their narratives, questions were asked to further explore the phases before, during, and after their home shifts. The interview guides from the individual interviews are appended.

The first author moderated the focus group interview (*n* = 4), with one assistant and three social educators supporting participants based on their individual needs, primarily related to signalling and turn‐taking. The interview lasted 78 min.

### 2.5 Analysis

**FIGURE 1:**
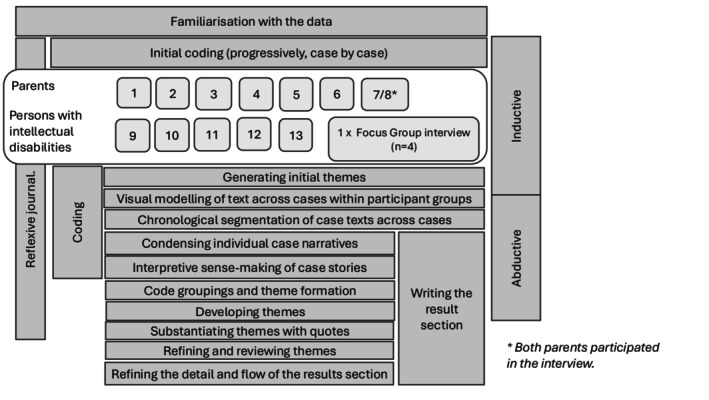
Overview of the analytical process.

All interviews were audio recorded and transcribed verbatim. The analysis began with familiarisation through listening to recordings, transcribing, and repeated reading of the entire dataset, along with notetaking of initial impressions (Braun et al. 2022; Braun and Clarke 2019, 2021, 2022, 2023). A broad overview of the analytical process is presented in Figure 1.

Aided by NVivo, the transcripts were initially coded case by case, progressively building on the expanding code structure. Initial themes were developed through immersion in the text and the interpretation of the underlying meanings of the codes and patterns across the transcripts within participant groups. Initial coding was conducted inductively, aiming to convey the meanings of the data and to depart from the participants' experiences, perspectives, and life‐worlds. Later stages of the coding and analysis treated experience as mediated and constructed through language, giving credence to the expectations, norms, values, and beliefs, as they relate to the social structures that encapsulate the phenomena under study, and progressed towards a more across‐case and abductive approach, where we drew on value co‐creation as a lens to interpret and make sense of the data.

The codes were grouped according to their common thematic connections. Subthemes were developed both within and across the participant groups. Themes were developed from the entire data set, integrating selections of subthemes and building from patterns of shared meaning and experience. Two phases significantly divided the data: the phase preceding the move, which was dominated by waiting, scarcity, and uncertainty about choice and rights, and the post‐move phase, marked by the unavoidable adjustment to others, both communally and socially, and more intimately. Themes were revised and refined during discussions with the co‐authors. The iterations of writing aided the tracking of the researchers' roles in the interpretative process of theme development, while ensuring coupling and alignment with the plain text and layers of meaning identified in and across the cases, both directly through quotes and indirectly through notes added to the reflexive journal. Table 2 presents an illustrative overview of an excerpt of the coding and analytical process.

**TABLE 2:** Illustrative overview of an excerpt of the codes, code groupings, subthemes, and themes.

Codes/code labels	Code groupings	Subthemes	Themes
− The municipality was slow − Little information on progress − Became a competition with other applicants − Other living arrangements was complicated − Did not know when or where to move − I waited for so long − I'd not understand the guidance − Nightshift was important for where to live − Expensive to buy and rent − Unclear who should do what …	Waiting Jealousy and unfairness Few options The first move Responsibility and rights …	A long, and increasingly uncertain search for an apartment Parents: Dejected and tattered, yet tethered to integrating resources Individuals: Nothing happened for long, then it went quick—from slow to go …	First, find a place

### 2.6 Reflexivity

The coding was conducted by the first author, a male Ph.D. candidate with over a decade of experience working in co‐located supported housing as an employee and administrative manager. Regarding personal reflexivity, the personal and professional values can, with some certainty, have influenced the analysis. In the reflexive journal there were also several notes related to the theoretical entry of value co‐creation and service theory and its impact on perspective on value, both what is seen and understood to be of value in a phenomenological sense, along with its broader dimensions and elements, and its creation process in public service settings (Cluley and Radnor 2021; Osborne 2020). The theoretical and disciplinary entry influenced interest in the service experience and bureaucratic manoeuvring. As the analysis description lays bare, there is a need to address functional reflexivity, as the design, methods, and analytical means can shape research and the knowledge produced (Braun and Clarke 2022). In the present study, both the narrative invitation in the interviews and the process‐tracing element are noteworthy. The first can influence the framing of the story and the information offered by participants, and the latter can influence the weight given to the segmentation of the sequential and temporal dimensions within experiential accounts, with an ever‐present risk of decoupling them from the whole life experience. Likewise, the decision to combine the two participant groups and develop themes across the entire dataset has material implications for the knowledge produced. It is self‐evident that the experiences themselves, during and after residential transitions, differ for the two participant groups; nonetheless, we believe that there are synergistic insights to be gleaned from the conjunction of the two participant groups, at least as they both pertain to the joint‐ and customer sphere and are public service user‐side experiences (Grönroos and Voima 2013; Osborne 2020).

### 2.7 Ethics

The study is approved by the Norwegian Agency for Shared Services in Education and Research (reference number: 101550). Efforts were made to reduce any risks and protect the rights of the participants and third persons. All participants received information about the study, including the option to withdraw at any time, in an easy‐to‐access format—both orally and in writing. Consent to participate and to be recorded during the interviews was obtained from all participants. All participants were given time to ask questions. Confidentiality was maintained by using codes and removing clearly identifiable details during transcription, in the presentation of quotes, and in reporting on participants' characteristics.

## 3 Results

From the analysis, two overarching themes were constructed to represent the experiences of adults with intellectual disabilities and parents: (1) ‘First, find a place’, and (2) ‘Settling in(to) a constructed home’. The themes are illustrated with verbatim participant quotes, annotated as follows: I# for persons with intellectual disabilities, P# for parent participants, and FG for excerpts from the focus group interview.

### 3.1 First, Find a Place

This theme captures a common experience among all participants' residential transition stories: long search phases driven by a scarcity of suitable housing. The accounts focus on interactions with, as well as the tempo and limitations of municipal services. The overall search processes were marked by uncertainty and escalating frustration. For the parent participants, there was an increasing exploration of alternatives and a growing realisation of the dynamics and importance of their role.

#### 3.1.1 A Prolonged and Uncertain Search

The extent of the participants' experiences with residential mobility varied, with just under half of the participants in both groups having only experienced the first move out of the family home. Adults with intellectual disabilities who had relatively recently moved out of the family home explained that moving out had been a recurring topic for years before the move. Several explained that it had been emotionally taxing to have the move hanging over them without a clear time or place. One participant stated:

It was very hard to talk about moving because I had never lived by myself before. Almost every time we talked about it, I was angry and sad. Mom said she would come and visit me often… but she also thought it was a little hard to talk about in a way. (I4)

Another participant recalled the wait similarly: ‘It was a very long process. I waited unbelievably long to get an apartment’ (I5). Yet when her offer finally arrived, she felt she had no real choice. Neither the location nor the support arrangement aligned with her wishes:

It was not to live in such an around‐the‐clock home I wanted. Because I wanted to live in a place where the staff just stops by, instead of being here all the time. So, I feel that my dream was crushed. And I did not, even if I attended the meetings about moving, get to decide how I would live. (I5)

Years later, she was still dissatisfied. Despite repeatedly stating her wish to move, nothing happened. When she raised the issue with support staff or in coordination meetings, they neither gave it the importance she felt it deserved nor followed up on it; instead, the discussion shifted to the extent of her support needs. Neither her economic guardian nor the few family members she still saw had the capacity to pursue the matter on her behalf. Finally, she stated:

I have lived here for seven years now, and this is not the place I want to live, but it is just impossible to get another place. (I5)

All parent participants also described long waiting times, noting additionally, particularly regarding the first move out of the family home, that they became increasingly aware of the situation's uncertainty as time passed. Several said they feared their care capacity would be reduced if other challenging life circumstances arose or the care burden increased, resulting in an acute and, perceived, even more difficult transition and suboptimal living arrangement. Several explained that they initially felt they could do little but meticulously document all aspects of their support needs to strengthen their case; yet, in retrospect, several wondered if these efforts mattered much in the end.

One parent explained that despite starting the process 4 years earlier, it all eventually went as she had feared, and her adult son had to be acutely relocated out of the family home. The parent described that it was all exacerbated by the pandemic, which had broken down their routine. Multiple episodes of very challenging behaviour the following year had upended everything, as she was left with no choice but to call the police to calm the situation. Notified of the development, the municipality could not provide any home‐based service to alleviate the matter, resulting in her adult son having to move out. Following several relocations between temporary facilities, the municipality announced a vacancy in a co‐located supported housing complex as the offered long‐term arrangement. The parent explained that they simply had to accept, because there were no other options. Two years after the move, the parent elaborated that both she and her adult son were very unhappy with his living arrangement. She highlighted the location, support regime, and, particularly, the staff logistics and institutional sphere around the home. The participant described that the search phase was, from her perspective, still ongoing, stating: ‘I have told him he will not live here for the rest of his life. You will get to move somewhere else, but it will take time because I must deal with the applications and stuff, but I don't know how much he understands’ (P3).

#### 3.1.2 Mounting Frustration

Parent participants described their efforts to prepare their adult child for moving and the central role they played in the search and the broader transition process. Many had explored purchasing or renting on the open market, sometimes in partnership with other families, but all were worried about the prolonged administrative burden and risks of arranging statutory support outside municipal schemes. All viewed, at least initially, municipal co‐located housing as the only realistic solution. Parents addressed how they were initially largely ignorant of any dynamics between their care capacity and the municipality's allocation logic and responsibility. One parent stated:

He lived at home until he was 27. Then we had passed 60 and we found that after giving our all night and day all these years, we were pretty spent and tired. There was a lot of grief associated with the decision to start looking for alternatives. … But it was hard to get the municipality to get going. (P8)

The municipality reinforced in its communication the perception that co‐located supported housing was the default option. This was further entrenched as some service elements, most frequently mentioned the access to on‐site night staff, were presented as dependent on residing in a co‐located setting: ‘… you could not get services if he did not live there. We just couldn't. Then we would lose the entire night shift’ (P2).

Applications were typically submitted when a child turned 16. One parent stated:

Every couple of years, we got asked if we still needed alternative living arrangements for her from the municipality. How is the situation? Do you need housing? Yes, we need supported housing. Then four or five years passed, and we got the same questions again. (P6)

Several described the municipality's waiting list as primarily a rhetorical construct, and that the allocation process was something they could not make much sense of and felt was unfair. One parent stated: ‘I said at one point that I request that my child be offered an apartment, given that we had been on the waiting list the longest of everybody now. That did not matter to them at all’ (P4).

Parents described the announced construction periods of new co‐located supported housing complexes as anticipation‐filled periods. Several parents recounted multiple projects that ended without them being offered an apartment, resulting in increased disappointment, frustration, and despair. Parents described increasingly experiencing a sense of competition with other waiting users and their families over the few available units that were announced.

After years of waiting, several parents described experiencing various inflexion points that altered their approaches. Accounting for an episode when she was notified that a forthcoming project had been cancelled due to lack of funding, one parent stated: ‘…I started to roll up my sleeves because then I realised that it would all rest on me, because the municipality obviously did not have any more responsibility’ (P2). Another parent explained that her approach changed when a long‐standing respite arrangement was cancelled due to an administrative error on her part: ‘But what does one do then? I mostly wanted to end it all and drive into the lake. … But then I thought, and maybe there was a little devil in me, I must get him an apartment and just notify them that he is living there by himself and let the municipality worry about getting him help’ (P4).

#### 3.1.3 From Slow to Go

Although the search phase often spanned years, once an offer for an apartment was obtained, the preparation, introduction, and acquainting with the residential care staff members, as well as the move itself, were recalled as a short and intense affair. One participant stated: ‘I did not know how it was going to be to live by myself, and when I got an apartment, it all happened very quickly, it went:—Smook! and I was living there’ (FG). Another participant said:

We visited before I moved. We went to a room that was completely empty. There was nothing there, and nobody was there, no table and no bed. So, we bought all the stuff we needed. A bed and different stuff. Then I moved …Everything was new, and then, many new people came that I had to spend time with. (I4)

Across accounts, the contrast between years of uncertainty and the swift tempo of the final move was striking. Once an apartment offer was obtained and accepted, an intense three‐month period unfolded to develop the services in preparation for moving in. After the run‐up phase, the accounts all described a brief and surprisingly regulated furnishing phase, followed by an extended and often quite turbulent settling‐in phase.

### 3.2 Settling In(to) a Constructed Home

This theme is constructed around the participants' accounts of the challenges they faced in settling into their new homes. Both participant groups experienced, albeit in different ways, the need to make various adjustments to their new surroundings and reported that the new living arrangement increasingly shaped their lives. The settling‐in phase was characterised by extensive changes in routines, roles, and the approaches to and objectives of the service offering. These changes, in turn, created tension about who had to adjust to whom and the balance and extent of agency.

#### 3.2.1 Adjusting to a Life Within the Scaffolding

Participants with intellectual disability often expressed that the move only became ‘real’ after the physical relocation. A common frustration was the large number of new people they had to build relationships with. The accounts further coalesced around the extent of change that ensued after the move, noting that it often entailed making adjustments that were initially experienced as illogical. One participant stated:

They weren't any good to guide me when I moved in, and a lot of stuff did not make any sense… I was supposed to learn to live alone, but the staff got mad at me if I locked my door. I was used to living with my mom in a house, and we had to lock the door every night. But when I did it, they came charging in and said: How in the world could I even think of locking my door?! (I2)

Further, changes in or termination of leisure activities following the move were particularly noted as unforeseen consequences and cited as additional burdens. One participant said: ‘I was in a choir… Then they told me it would be difficult because they did not have staff, because I needed to be chaperoned there…. When it comes to making stuff work for me, they are not very good’ (I5).

When it came to who had to adjust to whom, the participants with first‐hand moving experience stated that they often had to adapt. One participant said: ‘The first day I moved here, they took over the whole thing. So, that was something new to adjust to. It wasn't really any fun at all, because I was the one who had to get in line the whole time’ (I4). Another participant stated: ‘It was just a prison hole because the service decided everything about you’ (I2).

After the move, ‘the home’ increasingly became a factor in their accounts that one had to expend energy to become acquainted with, that one had to learn how to adjust to, that one often had to consult with, and which over time grew to become a constant fixture and intervening feature in their life. Contrastingly, there were also various nods and outright praise for the personal tailoring of the staff's support services during the settling‐in phase across the accounts. One participant stated: ‘Sometimes we think it's good to have the staff there, and sometimes we all find it a bit annoying, and sometimes we think they are a bit dumb’ (I4).

Several participants highlighted that as time went by after the move, they developed strong emotional bonds with select staff. Some accounts elaborated on the importance of these relationships, describing them in pseudo‐family terms, which also accounted for their growing emotional attachment and significance. One participant stated: ‘I now have a primary contact here that I am very fond of and that I have very close ties with’ (I5). The significance of this relationship was challenging to square with her general discontent with the living arrangement and her experience of the staff's exacerbated influence over her life.

Across the accounts of settling into their new home and the development of the support arrangement, some participants questioned the role, approaches, and objectives of the staff. Several noted that they often experienced tensions with the staff during the settling‐in phase. Some said that they had experienced new rules that were quite unilaterally introduced, often after an incident of some sort, for example, if they had tested out the bounds of their freedom by deciding to stay out late at night. One participant stated: ‘I wasn't really allowed to be out beyond the scheduled times’ (I2). The staff often developed new routines and practices incrementally, especially in the first period after they had moved in. Over time, participants reported that staff routines and practices could snowball, and consequently, their extent could be perceived as quite harsh and controlling. An example was the inquiry of where one was, who one was with, and when one was coming home. Over time, this could feel like a strict reporting regime, especially if the staff did not coordinate among themselves.

Several participants noted that the most challenging aspect after the move was establishing agreed‐upon terms with the staff regarding the extent of their roles and objectives, particularly concerning the limits these roles could impose on their agency and right to privacy in their home. One participant illustrated this tension when stating:

When I live here, I find it very difficult to have a boyfriend. Because every time I had him over, they would pop in and out to monitor what we were doing. I did not get any privacy, and I don't find that to be ok. (I5)

#### 3.2.2 Handing Over the Reins

The shift from the three‐month planning phase, centring on getting to know one another and transferring know‐how to construct routines, was marked across the parents' accounts. The settling‐in phase entailed that the parents experienced a shift in relevance, as they moved from being front‐stage and coordinating actors to finding themselves often increasingly sidelined and having to forge a new support role for themselves. This was frequently exacerbated by tension with management, particularly regarding logistics and staff selection, as well as disagreements with staff over approaches, objectives, and the execution of the service offering. Parents worked hard to convey their adult child's personality and life story. However, these efforts were rarely reflected in the services. One participant stated: ‘I feel like they took very little of what was part of our daily life into the system. So, when she moved, it seemed like everything from before was forgotten, and they were just set out to start over from scratch’ (P1).

Parents described that the approaches they had used prior often were plucked free from their life story context and stripped down to their bare logistical and actionable components. Often so much so that they felt the life of their adult child was timetabled and broken down into a list of chores.

Several noted that the large number of staff who rotated in was challenging. Beyond creating its own risk in terms of know‐how, it was emotionally and relationally straining to acquaint themselves with, transfer knowledge to, and interact with the large number of new staff. This consequently also impacted the emotional transition of letting go and finding a new role. One participant stated: ‘It isn't easy to let go when you feel that nobody is taking the reins’ (P1).

The parents acknowledged that the residential transition processes often introduced significant policy and system skepticism concerning the logistics, content, and approaches to the service offering. The prior acceptance of the care logic was often challenged throughout the process, particularly as they experienced limited influence and observed the impact of the living arrangement on their adult child's life. One parent stated:

They call it a collaboration meeting, but it is David that meets Goliath; they have, in most cases, already made up their minds, we get to talk some and state our opinions, but the decisions are already made. It is not a home they have been given; it is placed in an institution, and it is all run like an institution. (P5)

Parents found the settling‐in phase particularly challenging. It occurred at a time when the intensity of planning and the move had drained much of their energy, and the service indicated they were expected to start letting go. However, several explained that it was crucial to stay engaged even when the system attempted to detach them. Many noted that it was demanding to remain focused and involved so they could influence service development from the outset. One parent said: ‘The system is such that if you do not have strong family engagement, or rather, you need strong family engagement to get a good life’ (P1). Several pointed out that they increasingly became cognisant of the possibility that their family engagement could evolve towards becoming a respite and relief solution for the service, and that their involvement and resources became in essence, a failsafe for service quality shortcomings or even an opportunity for cost‐cutting rather than a contribution towards their adult child's quality of life. Another parent stated: ‘You get tired of constantly being on and checking up, but there is also a lot of grief attached to it, which demands a lot of energy by itself’ (P8). This ongoing vigilance and follow‐up on all relevant matters is described as a persistent struggle, compounded by the life sorrows that several reported as a constant emotional undercurrent in their lives.

## 4 Discussion

The findings illustrate that residential transitions for adults with intellectual disabilities and around‐the‐clock care needs, and their parents, involve more than a change of address. Rather, they unfold as extended and relational processes in which value is shaped across and beyond each phase of the residential transition. From a PSL perspective, these transitions involve a dynamic and interactive relationship in which value can be created or destroyed throughout the co‐creation process (Grönroos 2019; Grönroos and Voima 2013; Osborne et al. 2022). In line with previous research (Midttun et al. 2024; Mjøen 2019), we found that the home also functions as a service arena where relocation often disrupts established support systems, as staff rarely move with the individual. Beyond the first move out of the family home, residential transitions occur infrequently throughout most participants' lives; however, they are significant events with a consistent temporal rhythm. Across accounts, we identified three experiential structuring elements:

First, the scope of the statutory entitlement to services and their implications for housing were perceived as opaque. Distinct regulatory couplings between housing and services (Bendixen 2024) were unclear and largely eluded all participants.

Second, not only is the initial transition out of the family home often delayed (Roos and Søndenaa 2020), but subsequent planning, aspirations for future adult living arrangements, and opportunities for residential mobility are impeded relationally, structurally, and temporally by the immersive potential of service offerings and living arrangements.

Third, the length of the initial search phase may lead to strategic maneuvering in the case of the first move out of the family home. Over time, however, the experience of scarce housing options, the toll of engaging in the development of support services across and beyond residential transition processes, and more broadly, the unfolding reality of life in co‐located supported housing may lead to disillusionment with co‐located supported housing as a living arrangement.

Comparable to the prior literature on residential transitions (Roos and Søndenaa 2020), is the emergent temporality with significant tempo shifts between the long and uncertain waiting of the search phase, the abrupt, hasty planning and execution of the actual moving‐in phase, and the protracted period of adjustment that follows.

As illuminated in prior studies (McConkey et al. 2018; Roos and Søndenaa 2020; Sandvin and Anvik 2020), the ever‐looming possibility of an acute transition was both anticipated and, when it occurred, described by the parent group as stripping away almost all choice. Parents stated that they acted on their growing awareness of the dynamics between the limits of their parental responsibilities for caregiving and housing, and the municipality's responsibilities. An increased prevalence of urgent residential transitions has been reported (Astrup et al. 2019) and has been identified as a factor that complicates the application and allocation processes for the municipalities (Roos and Søndenaa 2020; Sandvin and Anvik 2020). The findings illustrate the impact it has on the process experiences of other waiting applicants.

The findings suggest that inadequate communication regarding the content and extent of rights, particularly the constitutive effects of the around‐the‐clock threshold derived from rights to services, influenced perceptions of the municipality's role, use of professional discretion, and responsibility. Limited understanding of the conditional nature of de facto responsibility could give rise to perceptions of malicious or unequal treatment, particularly among parents, and may contribute to further system estrangement and resignation among adults with intellectual disability. The perceived opacity surrounding housing‐related entitlements, especially when contrasted with the legally clearer and experientially more bureaucratically potent rights to services, shaped participants' perspectives on roles, responsibilities, and possibilities. The disanalogy contributed to a conflated perception of housing entitlements, exacerbating the expectations‐reality gap and, in turn, detracting from the search and residential transition experience. De facto, municipalities seek to construct economies of scale in service delivery. When combined with their responsibility to ensure adequate and coordinated care services, this has practical implications for the solutions offered when identifying viable options for care and housing as the preceptorial responsibility to provide shelter comes into effect because current living arrangements materially or functionally subside.

The participants described, in various ways, coping with the protracted search phase and the divergence from initial expectations as it unfolded. As similarly reported by Jacobs et al. (2021), the volume and turnover of staff made settling in particularly challenging. Towards the later stages of residential transition processes, parents pointed to the lack of trust as a significant impediment to letting go, a point also highlighted in a Dutch study by Vereijken et al. (2024). However, in our findings, this extended well beyond any individual service provider or the staff group in general, and instead encompassed the nature, capabilities, and limits of co‐located supported housing. Parents reported a growing realisation that their continual involvement was paramount to safeguarding agency, identity, and quality of life, as well as to mitigate service delivery risks and dislodge their adult offspring's total life embedding in the institutional frame of the service setting. Our findings further echo elements of the work of Codd and Hewitt (2021) and Skagestad et al. (2023), given the reported uneasy balance between parents' participant roles, their modes of engagement with the public service provider, and their long‐term role, in our case, especially related to bearing much of the burden of facilitating future residential mobility.

The findings illustrate that persons with intellectual disabilities experience significant power asymmetries across the residential transition processes, both in having their voice heard in the search phase and in relation to having any real choice once an offer was finally on the table. In line with previous research, participants experienced a decoupling between talk and practice after the transition, as the professed intent of developing independence and self‐determination had to be negotiated against the permeating institutional frames of their new home and the role of the service staff in relation to privacy and control over their lives (Bigby et al. 2017; Midttun et al. 2024). In accordance with Guddingsmo (2019), our findings illustrate how persons adjust to and may become dependent on their living arrangements, and indicate that the bonds with key care staff can become a relational impediment to future residential mobility.

The findings of this study substantiate prior Norwegian research that has shown that the search for suitable housing often spans years (Roos and Søndenaa 2020; Sandvin and Anvik 2020). They further indicate that waiting times are even longer, and challenges are more pronounced in cases where the extent of individuals' around‐the‐clock needs was not overtly high or readily recognised in relation to the threshold of around‐the‐clock service needs. In situations where an individual sought to relocate based on their own volition while residing in co‐located supported housing, a state of prolonged liminality could emerge. Our findings show that adults with intellectual disabilities often experience helplessness and resignation. Persons with intellectual disabilities reported perceptions of confinement, both geographically due to the limited options available once they finally moved, and in terms of service level and form of living arrangement. Several noted that their needs, actual abilities, preferences—particularly regarding privacy—and aspirations for independence evolved. However, they found their opportunities to move to be dependent on others and generally viewed their chances for residential mobility as bleak.

## 5 Implications for Practice, Policy and Theory

The findings suggest that residential transitions to and within co‐located supported housing should be understood as extended processes rather than discrete housing events. For practice, this points to the importance of continuity across the transition trajectory, including clearer communication during the search phase, better preparation before the move, and follow‐up beyond the settling‐in phase. The findings also indicate that what happens after the move matters greatly for how the new living arrangement is experienced in everyday life. Practice may therefore benefit from greater attention to continuity, coordination, and clarification of roles and expectations before and after relocation. For parents, recalibrating involvement into new forms may be particularly challenging. This suggests a need to recognise family involvement as a resource in the longer transition process, while also ensuring that responsibility is not shifted onto families in ways that primarily function to relieve the municipal service system.

At the policy level, the findings point to the need for clearer communication about housing‐related rights, eligibility thresholds, and the practical relationship between housing and services. Participants were often uncertain about what the municipality was obliged to provide, what depended on professional discretion, and why access to particular forms of support seemed tied to specific housing arrangements. In practice, this uncertainty appears to narrow the scope of meaningful choice and contribute to disappointment, mistrust, and reduce confidence in future mobility. The findings also suggest that rights‐based expectations may exceed the reality of individually enforceable rights. In the Norwegian context, recent legal and policy discourse on the CRPD (UN 2006) indicates that incorporation of the Convention into Norwegian law does not constitute new rights beyond those already grounded in domestic law. The practical realisation of, for example, Article 19 therefore remains mediated by municipal discretion and available resources (The Royal Ministry of Justice and Public Security 2025, 47–48). Against this backdrop, participants' experiences depended heavily on how municipalities interpreted, communicated, and operationalised existing rights and responsibilities. A key policy implication is therefore not only the need for more housing options, but also for more transparent communication about criteria, processes, and realistic possibilities for relocation over time. This may contribute to reducing disillusionment and better align rights‐based expectations with service realities, even where actual mobility remains limited.

Theoretically, the findings indicate that value creation is not confined to the move itself or to discrete moments of service delivery. Instead, it unfolds across connected phases of searching, moving, and settling in, and is shaped by the interactions among people with intellectual disabilities, parents, staff, service managers, housing arrangements, rules, and municipal conditions. Participants did not encounter these service arrangements only in material and organisational terms. They also experienced them through expectations, hopes, disappointments, trust, perceptions of fairness, and judgements about what the home could become. This resonates with Cluley and Radnor's (2021) argument that co‐creation in public services should be understood as fluid, relational, and shaped by elements beyond a simple two‐way interaction. It also aligns with Strokosch and Osborne's (2020) ecosystem perspective, and with the broader turn towards a public service ecosystem framework within PSL (Osborne et al. 2022; Trischler et al. 2023), which emphasises that value creation is enabled and constrained by wider participatory, organisational, institutional, and sub‐micro conditions, including actors' beliefs, expectations, and judgements. The findings thus suggest that participation and choice are not stable preconditions for value creation, but contingent accomplishments that may be narrowed by scarcity, professional discretion, organisational routines, the coupling of housing and support services, and by how actors interpret and evaluate the service experience over time (Osborne 2018). The study adds to PSL by illustrating how the wider service ecosystem can shape value‐for‐whom across time and contribute not only to value co‐creation, but also to disillusionment, reduced agency, and value co‐destruction. Furthermore, it extends the literature on value creation involving vulnerable users, a domain that remains notably underexplored (Osborne 2018).

## 6 Limitations

Although the sample is considered trustworthy, several important limitations of this study warrant mention. The study is based on a relatively small number of interviews, and all participants either live in, have previously lived in, or, for the parent group, have their adult children residing in municipality‐operated, 24‐h‐staffed, co‐located supported housing. Moreover, all persons with intellectual disabilities resided in supported housing and/or received services from the same average‐sized municipality in a semi‐rural area of Norway.

Drawing on the municipality's network was the main component of the recruitment strategy. Nonetheless, despite efforts to broaden recruitment to include more parent–child dyads and participants with more recent experiences and a broader range of functioning, the sample across both groups consisted mainly of women, and only half had ongoing processes or had their most recent home‐shift experiences within the last 5 years.

Our data consisted of only one parent–child dyad, and the approach privileges the independent accounts of the two participant groups regarding process and actor focus. A dyadic design would likely be better suited to highlight the relational and actor dynamics as factors within the broader experience of residential transitions for the two participant groups.

Approximately half of the data consisted of interviews comprised of stories, reflections, and answers from people with intellectual disabilities, with varying communication and language skills. This necessitated both more straightforward evaluations and patch‐making to elaborate experiences and contextualise events. The possibility of social desirability bias derived from the questioning and inadequate support should also be considered.

## 7 Conclusion

Residential transitions to and within co‐located supported housing are best understood not as discrete housing events, but as extended and relational public service processes shaped by prolonged waiting, limited choice, abrupt tempo shifts, and often challenging post‐move adjustments. From a PSL perspective, value creation in these transitions unfolds across the connected phases of searching, moving, and settling in, and may result in both value co‐creation and value co‐destruction.

The findings suggest that these transitions affect more than the move itself. They shape how adults with intellectual disabilities and parents perceive choice and construct understandings of the constraints on future residential mobility. Negative experiences in the search phase, the move itself, and the development of support services may reduce trust, constrain agency, and diminish aspirations to relocate to a more suitable living arrangement in the future.

For both adults with intellectual disabilities and parents, the findings point to persistent uncertainty about rights, responsibilities, and realistic opportunities for mobility. They also suggest that the way municipalities communicate roles, responsibilities, and explain the relationship between housing and services matters for how transition processes are experienced. In this respect, the study points to a gap between policy and de facto practice, as well as between participants' expectations and the practical realities of housing entitlements and service provision.

A central implication is therefore the need for clearer communication about housing‐related rights, more explicit articulation of criteria and processes, and greater attentiveness to mobility beyond the first move. Such measures may reduce disillusionment, better align expectations with service realities, and improve experiences of residential transition processes, even where actual mobility remains limited.

## Author Contributions

Conceptualisation: K.U., L.T., K.A. and S.F.M. Methodology: K.U., L.T., K.A. and S.F.M. Analysis: K.U., K.A., L.T. and S.F.M. Data collection: K.U. Data curation: K.U. Writing – original draft preparation: K.U. Writing – review and editing: K.A., L.T. and S.F.M. Supervision: S.F.M, L.T. and K.A. Project administration: K.U. All authors have read and agreed to the published version of the manuscript. Position of authorship has been agreed.

## Funding

The authors have nothing to report.

## Conflicts of Interest

The authors declare no conflicts of interest.

## Data Availability

Research data are not shared.

## Appendix A Semi‐Structured Interview Guide—Persons With Intellectual Disabilities and Firsthand Moving Experience

Excerpt of the question component:

### Opening Questions

Could you tell me a little about yourself?

− How old are you?
− What do you like to do?
− How is a typical weekday for you?
− What do you like to do on the weekend?

### Guiding Questions

1. Can you tell the story about when you moved for the first time?
2. Can you tell me about when you moved here?
3. What happened before you moved in?
4. What happened after you moved in?
5. Have you moved before?
6. What happened then?
7. What is important to you in your home?
8. What do you think about moving in the future?

## Appendix B Semi‐Structured Interview Guide—Parents

Excerpt of the question component:

### Opening Questions

Before we start to talk about your experiences with residential transitions for your son/daughter, could you tell me a little about yourself and your family?

− Where are you from?
− What do you do?
− How is your family situation?

If it is okay with you, I would like to start by asking you to tell me a bit about yourself, your role as a next of kin, and your experiences.

### Guiding Questions

1. Could you tell me the story about when X moved (the first time, n, last time)?
2. What were your expectations related to the move, and how did you experience the move?
3. When did the process of finding a new living arrangement for *NAME* start, and how did that unfold?
4. Looking back at the last move, what was positive about the experience?
5. From your perspective, what could have been better?
6. What do you think about *NAME* moving in the future?

### Segments of Follow‐Up Questions

#### Alternative Living Arrangements

Did *NAME* or you consider other forms of housing?

- Were you given information and guidance about alternative housing options?
- Did you consider other forms of housing (owning, renting, co‐located housing, etc.)?
- How do you experience the work being done to facilitate housing options other than co‐located housing?
- What do you think was decisive for the choice of living arrangement/housing solution?

#### Application and Planning Related to Housing (and Services)

Did *NAME* or you apply for any public services?

- Did *NAME* or you apply for housing bank loans or other financial aid schemes?
- How did the application process proceed, and how did you experience it?
- Did you have any prior knowledge of or experience with housing/service applications?

#### Housing and Services

Did you experience a link between housing and services?

- Do you experience that the housing situation is decisive for the scope or quality of the services that *NAME* receives?
- Do you experience that consideration for other co‐located service users affects the scope or quality of the services that *NAME* receives?

#### Influence, Information and Collaboration

Can you share some experiences from your collaboration with the municipality?

- In what way were you involved, and how has this involvement taken place? Please feel free to describe with a few short examples.
- Are there any requirements for you to participate, or is it expected that you should be active in planning and designing services?
- To what extent are you required to participate?
- To what extent do you demand participation yourself?
- Are you, as a next of kin, asked to provide information about *NAME*?
- Do you feel that you were involved at the right time?
- What were your expectations of the process?
- Did you experience any attempts to manage or adjust your expectations regarding the housing or services for *NAME*?

#### Co‐Creation

What do you understand by the term *co‐creation*?

- What does it mean to you?
- How did you experience that co‐creation was facilitated when NAME moved?

#### Family Participation

How important do you think it is that you have influence and the opportunity to participate in the preparation to move and the design of services?

- Are there factors that prevent you, as a next of kin, from participating?
- Who do you need to collaborate with?
- Have you experienced resistance from staff related to your or the service user's participation?
- Have decisions been made or services designed that you believe could have been much better if you, as a next of kin, had been more involved?
- Have you been presented with proposals involving changes to the service user's services that you disagreed with?
- Have there been challenging situations or disagreements in negotiations regarding housing or services? How did these arise, and how were they addressed or handled?
- What significance does participation have for the content and quality of the services?
- Do you experience alignment between your views and *NAME's* needs?

#### NAME Involvement

How do you experience *NAME's* involvement in the home shift?

- Are there factors that prevent *NAME* from participating?
- Would it be possible to better facilitate *NAME's* participation in the move and service development?

#### Role of the Next of Kin

What kind of work do you do as a next of kin?

- What role did you play in the move?
- Is what you do something you want to do, or would you prefer that someone else did it?
- What expectations do staff have of you, and how do you experience these expectations?
- What expectations do you have of yourself?
- Do you experience any negative aspects related to your participation or involvement?

#### Being Heard

Do you feel that you were/are being heard?

- How do you experience communication with the municipality?
- Do you feel that the information you receive is understandable and useful?
- Is it important to you or to NAME who the others living in the co‐located supported housing are?
- Have you had any influence on who *NAME* lives close to?

#### Value and Quality

What creates a good home and good services for *NAME*?

- What creates value for you?
- What creates value for *NAME*?

#### Current Housing Situation and Future Mobility

What is happening in *NAME's* life in relation to housing now?

- What is important to *NAME* when it comes to housing?
- What is important to you/you all when it comes to housing for *NAME*?
- Are there any plans to move for *NAME* now/in the future?

### Closing

We have now talked about various issues related to your experiences with the residential transition and service development for NAME.

Is there anything else you would like to add that I have not asked you about?

## References

[jar70225-bib-0001] Act Relating to Social Services . 2009. “Lov om Sosiale Tjenester i Arbeids‐ og Velferdsforvaltningen (Sosialtjenesteloven): LOV‐2009‐12‐18‐131.” Lovdata. https://lovdata.no/dokument/NL/lov/2009‐12‐18‐131.

[jar70225-bib-0002] Astrup, K. C. , R. Barlindhaug , and M. E. Ruud . 2019. “Boligeie for Personer Med Utviklingshemming—Utfordringer, Effekter og Incentiver (NIBR‐Rapport 2019:15).” https://biblioteket.husbanken.no/arkiv/dok/Komp/Boliger%20for%20personer%20med%20utviklingshemming.pdf.

[jar70225-bib-0003] Bendixen, A. 2024. “Inngripende Vilkårsstillelse i Tilrettelagt, Kommunal Bolig.” Kritisk Juss 50, no. 1: 1–27. 10.18261/kj.50.1.1.

[jar70225-bib-0004] Bennett, A. , and J. T. Checkel . 2015. Process Tracing: From Metaphor to Analytic Tool. Cambridge University Press.

[jar70225-bib-0005] Berlin Hallrup, L. , C. Kumlien , and E. Carlson . 2019. “Service Managers' Experiences of How the Participation of People With Intellectual Disabilities Can Be Promoted in Swedish Group Homes.” Journal of Applied Research in Intellectual Disabilities 32, no. 2: 427–434. 10.1111/jar.12540. 30453384

[jar70225-bib-0006] Bigby, C. , E. Bould , and J. Beadle‐Brown . 2017. “Conundrums of Supported Living: The Experiences of People With Intellectual Disability.” Journal of Intellectual & Developmental Disability 42, no. 4: 309–319. 10.3109/13668250.2016.1253051.

[jar70225-bib-0007] Braun, V. , and V. Clarke . 2019. “Reflecting on Reflexive Thematic Analysis.” Qualitative Research in Sport, Exercise and Health 11, no. 4: 589–597. 10.1080/2159676X.2019.1628806.

[jar70225-bib-0008] Braun, V. , and V. Clarke . 2021. “One Size Fits All? What Counts as Quality Practice in (Reflexive) Thematic Analysis?” Qualitative Research in Psychology 18, no. 3: 328–352. 10.1080/14780887.2020.1769238.

[jar70225-bib-0009] Braun, V. , and V. Clarke . 2022. Thematic Analysis: A Practical Guide. SAGE.

[jar70225-bib-0010] Braun, V. , and V. Clarke . 2023. “Toward Good Practice in Thematic Analysis: Avoiding Common Problems and Be(Com)ing a *Knowing* Researcher.” International Journal of Transgender Health 24, no. 1: 1–6. 10.1080/26895269.2022.2129597. 36713144 PMC9879167

[jar70225-bib-0011] Braun, V. , V. Clarke , and N. Hayfield . 2022. “‘A Starting Point for Your Journey, Not a Map’: Nikki Hayfield in Conversation With Virginia Braun and Victoria Clarke About Thematic Analysis.” Qualitative Research in Psychology 19, no. 2: 424–445. 10.1080/14780887.2019.1670765.

[jar70225-bib-0012] Brinkmann, S. , and S. Kvale . 2015. InterViews: Learning the Craft of Qualitative Research Interviewing. 3rd ed. Sage.

[jar70225-bib-0013] Clark, T. , L. Foster , L. Sloan , A. Bryman , and T. Clark . 2021. Bryman's Social Research Methods. 6th ed. Oxford University Press.

[jar70225-bib-0014] Cluley, V. , and Z. Radnor . 2021. “Rethinking Co‐Creation: The Fluid and Relational Process of Value Co‐Creation in Public Service Organizations.” Public Money & Management 41, no. 7: 563–572. 10.1080/09540962.2020.1719672.

[jar70225-bib-0015] Codd, J. , and O. Hewitt . 2021. “Having a Son or Daughter With an Intellectual Disability Transition to Adulthood: A Parental Perspective.” British Journal of Learning Disabilities 49, no. 1: 39–51. 10.1111/bld.12327.

[jar70225-bib-0016] Cui, T. , and S. P. Osborne . 2022. “Unpacking Value Destruction at the Intersection Between Public and Private Value.” Public Administration 101, no. 4: 1207–1226. 10.1111/padm.12850.

[jar70225-bib-0017] Dommermuth, L. 2009. “Når Flytter de Unge Hjemmefra?: Utflytting Fra Oppveksthjemmet. [When Do the Young People Move Away From Home?: To Leave Upbringing Home] (No. 1/2009; Samfunnsspeilet).” 9–12. https://www.ssb.no/befolkning/artikler‐og‐publikasjoner/naar‐flytter‐de‐unge‐hjemmefra.

[jar70225-bib-0018] Ferraretto, V. , and A. Vitali . 2023. “Parental Socioeconomic Status and Age at Leaving Home in Europe: Exploring Regional Differences.” Population, Space and Place 29, no. 6: e2679. 10.1002/psp.2679.

[jar70225-bib-0019] Grey, J. M. , G. M. Griffith , V. Totsika , and R. P. Hastings . 2015. “Families' Experiences of Seeking Out‐Of‐Home Accommodation for Their Adult Child With an Intellectual Disability: Families' Experiences of Seeking Accommodation.” Journal of Policy and Practice in Intellectual Disabilities 12, no. 1: 47–57. 10.1111/jppi.12106.

[jar70225-bib-0020] Grönroos, C. 2019. “Reforming Public Services: Does Service Logic Have Anything to Offer?” Public Management Review 21, no. 5: 775–788. 10.1080/14719037.2018.1529879.

[jar70225-bib-0021] Grönroos, C. , and P. Voima . 2013. “Critical Service Logic: Making Sense of Value Creation and Co‐Creation.” Journal of the Academy of Marketing Science 41, no. 2: 133–150. 10.1007/s11747-012-0308-3.

[jar70225-bib-0022] Guddingsmo, H. 2019. “‘Da Må Jeg Spørre Boligen Først!’—Opplevelsen av Selvbestemmelse i Bofellesskap.” In Hverdag i Velferdsstatens Bofellesskap, edited by J. Tøssebro , 78–93. Universitetsforlaget.

[jar70225-bib-0023] Health and Care Services Act . 2011. Lov om Kommunale Helse‐ og Omsorgstjenester m.m. (Helse‐ og Omsorgstjenesteloven) (LOV‐2011‐06‐24–30). Lovdata. https://lovdata.no/dokument/NL/lov/2011‐06‐24‐30.

[jar70225-bib-0024] Jacobs, P. , E. Quayle , H. Wilkinson , and K. MacMahon . 2021. “Relationships Matter! —Utilising Ethics of Care to Understand Transitions in the Lives of Adults With Severe Intellectual Disabilities.” British Journal of Learning Disabilities 49, no. 3: 329–340. 10.1111/bld.12380.

[jar70225-bib-0025] Langley, A. 1999. “Strategies for Theorizing From Process Data.” Academy of Management Review 24, no. 4: 691. 10.2307/259349.

[jar70225-bib-0026] McConkey, R. , F. Kelly , S. Craig , and F. Keogh . 2018. “Irish Persons With Intellectual Disability Moving From Family Care to Residential Accommodation in a Period of Austerity.” Journal of Applied Research in Intellectual Disabilities 31, no. 5: 833–839. 10.1111/jar.12439. 29424014

[jar70225-bib-0027] Midttun, A. L. , A. Gjermestad , and I. M. Lid . 2024. “Moving Out to Live Independently? Experiences From Young Women With Intellectual Disabilities in Norway.” Nordic Social Work Research 15: 1–13. 10.1080/2156857X.2024.2318594.

[jar70225-bib-0028] Mjøen, O. M. 2019. Å Arbeide i Noens Hjem. [Doktoravhandling]. NTNU.

[jar70225-bib-0029] Osborne, S. P. 2018. “From Public Service‐Dominant Logic to Public Service Logic: Are Public Service Organizations Capable of Co‐Production and Value Co‐Creation?” Public Management Review 20, no. 2: 225–231. 10.1080/14719037.2017.1350461.

[jar70225-bib-0030] Osborne, S. P. 2020. Public Service Logic: Creating Value for Public Service Users, Citizens, and Society Through Public Service Delivery. 1st ed. Routledge. 10.4324/9781003009153.

[jar70225-bib-0031] Osborne, S. P. , M. Powell , T. Cui , and K. Strokosch . 2022. “Value Creation in the Public Service Ecosystem: An Integrative Framework.” Public Administration Review 82, no. 4: 634–645. 10.1111/puar.13474.

[jar70225-bib-0032] Østenstad, B. H. 2011. “En Studie av Gjeldende Rett Etter Lov om Sosiale Tjenester m.v. (§§ 3–4, 4–2 Bokstav d), jf. 4‐3 og Lov om Sosiale Tjenester i Arbeids‐ og Velferdsforvaltningen § 15 i Lys av Norges Menneskerettslige Forpliktelser.” https://bora.uib.no/bora‐xmlui/handle/1956/5454?locale‐attribute=en.

[jar70225-bib-0033] Patients and User Rights Act . 1999. “Lov om Pasient‐ og Brukerrettigheter (Pasient og Brukerrettighetsloven) (LOV‐1999‐07‐02‐63).” *Lovdata.no*. https://lovdata.no/dokument/NL/lov/1999‐07‐02‐63.

[jar70225-bib-0034] Prop. 99 L (2015–2016) . 2016. “Proposisjon Til Stortinget 99 (2015–2016), Endringer i Pasient‐ og Brukerrettighetsloven og Helse‐ og Omsorgstjenesteloven. Helse‐ og Omsorgsdepartementet.” https://www.regjeringen.no/no/dokumenter/prop.‐99‐l‐20152016/id2483443/.

[jar70225-bib-0035] Roos, E. , and E. Søndenaa . 2020. “Improving the Transition Process to Independent Living for Adolescents With Profound Intellectual Disabilities. Experiences of Parents and Employees.” BMC Health Services Research 20, no. 1: 1133. 10.1186/s12913-020-05976-y. 33298053 PMC7724626

[jar70225-bib-0036] Sandvin, J. T. , and C. H. Anvik . 2020. “Tjenester Til Personer Med Utviklingshemming—i Spenningen Mellom ny og Gammel Omsorgsideologi.” In Velferdstjenestenes Vilkår, 67–89. Universitetsforlaget. 10.18261/9788215034713-2020-5.

[jar70225-bib-0037] Šiška, J. , and J. Beadle‐Brown . 2023. “Progress on Deinstitutionalisation and the Development of Community Living for Persons With Disabilities in Europe: Are We Nearly There?” Disability & Society 38, no. 8: 1476–1495. 10.1080/09687599.2022.2071676.

[jar70225-bib-0038] Skagestad, L. J. , S. Østensjø , and O. S. Ulvik . 2023. “Young Adults With Disabilities and Their Transitions to Adult Life and Services: A Sociocultural Analysis of Parents' Perspectives on Their Involvement.” Scandinavian Journal of Disability Research 25, no. 1: 106–118. 10.16993/sjdr.890.

[jar70225-bib-0039] Stake, R. E. 1995. The Art of Case Study Research. Sage Publications.

[jar70225-bib-0040] Strokosch, K. , and S. P. Osborne . 2020. “Co‐Experience, Co‐Production and Co‐Governance: An Ecosystem Approach to the Analysis of Value Creation.” Policy & Politics 48, no. 3: 425–442. 10.1332/030557320X15857337955214.

[jar70225-bib-0041] The National Board of Health and Welfare . 2021. “Insatser Och Stöd Till Personer Med Funktionsnedsättning. Lägesrapport (Service and Support for People With Disabilities. Current Status Report).” https://www.socialstyrelsen.se/globalassets/sharepoint‐dokument/artikelkatalog/ovrigt/2021‐3‐7327.pdf.

[jar70225-bib-0042] The Norwegian Directorate of Health . 2021. “Nasjonal Veileder: Gode Helse‐ og Omsorgstjenester Til Personer Med Utviklingshemming.” https://www.helsedirektoratet.no/veiledere/gode‐helse‐og‐omsorgstjenester‐til‐personer‐med‐utviklingshemming.

[jar70225-bib-0043] The Royal Ministry of Justice and Public Security . 2025. “Prop. 162 L (2024–2025) Endringer i Menneskerettsloven mv.—(Inkorporering av FN‐Konvensjonen om—Rettighetene Til Mennesker Med Nedsatt—Funksjonsevne).” https://www.regjeringen.no/no/dokumenter/prop.‐162‐l‐20242025/id3116949/.

[jar70225-bib-0044] The Social Housing Act . 2022. Lov om Kommunenes Ansvar på Det Boligsosiale Feltet [Boligsosialloven] (LOV‐2022‐12‐20‐121). Lovdata. https://lovdata.no/dokument/NL/lov/2022‐12‐20‐121.

[jar70225-bib-0045] Tøssebro, J. 2016. “Scandinavian Disability Policy: From Deinstitutionalisation to Non‐Discrimination and Beyond.” Alter 10, no. 2: 111–123. 10.1016/j.alter.2016.03.003.

[jar70225-bib-0046] Tøssebro, J. 2019. Hverdag i Velferdsstatens Bofellesskap. Universitetsforlaget.

[jar70225-bib-0047] Tøssebro, J. , I. S. Bonfils , A. Teittinen , M. Tideman , R. Traustadóttir , and H. T. Vesala . 2012. “Normalization Fifty Years Beyond‐Current Trends in the Nordic Countries: Normalization Fifty Years Beyond.” Journal of Policy and Practice in Intellectual Disabilities 9, no. 2: 134–146. 10.1111/j.1741-1130.2012.00340.x.

[jar70225-bib-0048] Tøssebro, J. , and C. Wendelborg . 2021. “Utviklingshemmetes Bosituasjon 2021 (Mangfold og Inkludering) [NTNU Samfunnsforsking AS, Mangfold og Inkludering].”

[jar70225-bib-0049] Trischler, J. , M. Røhnebæk , B. Edvardsson , and B. Tronvoll . 2023. “Advancing Public Service Logic: Moving Towards an Ecosystemic Framework for Value Creation in the Public Service Context.” Public Management Review 1–29: 1–29. 10.1080/14719037.2023.2229836.

[jar70225-bib-0050] UN . 2006. The Convention of the Rights of People With Disabilities. United Nations CRPD.

[jar70225-bib-0051] Vereijken, F. R. , S. A. H. Giesbers , A. Jahoda , and P. J. C. M. Embregts . 2024. “The Experiences of Parents Arranging the Move of Their Young Adult Offspring With Intellectual Disabilities to 24‐Hour Residential Settings; a Continuing Puzzle.” Journal of Intellectual & Developmental Disability 49, no. 3: 298–310. 10.3109/13668250.2023.2254942. 39815960

[jar70225-bib-0052] Walker, R. , I. Belperio , C. Bigby , I. Wiesel , F. Rillotta , and C. Hutchinson . 2025. “The Transition From Family Home to Alternative Living Arrangements: Experiences of Adults With Intellectual Disabilities and Their Family Members.” Journal of Applied Research in Intellectual Disabilities 38, no. 2: e70047. 10.1111/jar.70047. 40183308 PMC11969629

[jar70225-bib-0053] Zaviršek, D. , and S. Fischbach . 2023. “Independent Living in Post‐Socialist Countries: Between Familialism, Deinstitutionalisation, and Reinstitutionalisation.” International Journal of Disability and Social Justice 3, no. 1: 96. 10.13169/intljofdissocjus.3.1.0096.

